# Standardised assessment of patients' capacity to manage medications: a systematic review of published instruments

**DOI:** 10.1186/1471-2318-9-27

**Published:** 2009-07-13

**Authors:** Rohan A Elliott, Jennifer L Marriott

**Affiliations:** 1 Centre for Medicine Use and Safety, Faculty of Pharmacy and Pharmaceutical Sciences, Monash University, Parkville, Victoria 3052, Australia; 2 Pharmacy Department, Austin Health, Studley Rd, Heidelberg, Victoria 3084, Australia

## Abstract

**Background:**

Older people are commonly prescribed complex multi-drug regimens while also experiencing declines in the cognitive and physical abilities required for medication management, leading to increased risk of medication errors and need for assisted living. The purpose of this study was to review published instruments designed to assess patients' capacity to self-administer medications.

**Methods:**

Searches of Medline, EMBASE, CINAHL, PsycINFO, International Pharmaceutical Abstracts, Health and Psychosocial Instruments, Google, and reference lists of identified publications were conducted to identify English-language articles describing development and validation of instruments designed to assess patients' capacity to self-administer medications. Methodological quality of validation studies was rated independently against published criteria by two reviewers and reliability and validity data were reviewed.

**Results:**

Thirty-two instruments were identified, of which 14 met pre-defined inclusion criteria. Instruments fell into two categories: those that used patients' own medications as the basis for assessment and those that used a simulated medication regimen. The quality of validation studies was generally low to moderate and few instruments were subjected to reliability testing. Most instruments had some evidence of construct validity, through associations with tests of cognitive function, health literacy, activities of daily living or measures of medication management or adherence. Only one instrument had sensitivity and specificity data with respect to prediction of medication-related outcomes such as adherence to therapy. Only three instruments had validity data from more than one independent research group.

**Conclusion:**

A number of performance-based instruments exist to assess patients' capacity to manage their own medications. These may be useful for identifying physical and cognitive barriers to successful medication management, but further studies are needed to determine whether they are able to accurately and reliably predict medication outcomes.

## Background

Older people are commonly prescribed complex multi-drug regimens, while also experiencing declines in the cognitive and physical abilities required for medication management [1,2]. Reduced competence in medication self-administration is associated with higher risk of hospitalisation and requirement for assisted living [3-5].

Health professionals are not good at predicting patients' ability to perform instrumental activity of daily living (IADL), and impaired functional ability may not be detected in routine clinical examinations [6]. More accurate and reliable assessment methods are required [6]. Because high-level IADLs such as medication management are dependent on cognitive abilities, global measures of cognitive function such as the mini-mental state examination (MMSE) are sometimes used to predict functional ability. However, these measures have demonstrated inconsistent relationships with functional performance and lack sensitivity in discriminating competent from incompetent individuals [2,7]. Most measures of IADL performance rely on self-report or informant report, which is a reasonable starting point since it is relatively quick and easy [2,6]. However, a limitation is that these measures are subjective and prone to bias [6]. Some older people have difficulty evaluating their own competence or are reluctant to admit their inability to cope, leading to over-estimation of performance [6,8]. Family members or caregivers may under- or over-estimate performance, or may be unable to make a determination because of a lack of opportunity to observe the patient [6,8]. Furthermore, IADL scales are usually limited by reliance on a single item to assess functional ability within complex domains such as medication management and lack of well-defined definitions for the skill required [8]. They also provide little information as to the cause of the incapacity. Sometimes health professionals judge IADL performance based on informal observation of the patient, but these unstructured assessments are of unproven efficiency for predicting performance, and the results are usually unquantified and therefore not amenable to comparison across or within individual patients [9].

There has been increasing interest in recent years in developing standardised, structured, performance-based measures that address the high-level IADLs using materials that patients actually encounter in their daily lives [8,10]. Such instruments can provide useful information not only in terms of a score but also in terms of qualitative data relating to the individual's specific deficits.

The ideal assessment instrument should be objective, quantitative, reliable, readily administered with minimal training, easily and immediately scorable, brief, small and portable and non-threatening to the subject. It should assess both cognitive and physical abilities, [2,11] and demonstrate clinical utility by providing information to guide planning, application and/or monitoring of medication management interventions. It should have better (or more reliable) predictive validity than self-report, informant report and non-standardised assessment, and demonstrate sensitivity to changes in function over time as cognitive or physical function deteriorates.

A recent review identified 16 instruments designed to assess patients' capacity to manage medications [12]. However the review was limited by the omission of several instruments and validation studies, and the absence of critical appraisal of the methodological quality of validation studies. The objective of our study was to critically review the reliability and validity of published instruments designed to assess patients' capacity to self-administer medications and identify those that may be suitable for use in clinical practice or research.

## Methods

### Search strategy

A search of Medline (1966–2007), EMBASE (1980–2007), CINAHL (1982–2007), PsycINFO (1967–2007) International Pharmaceutical Abstracts (IPA; 1970–2007) and Health and Psychosocial Instruments (1985–2007) was conducted using the following terms: self administration, medication, medicine or drug, and ability, capacity, competence, skill or geriatric assessment (limited to English language). Relevant reference texts [13-15], and Internet sites [16,17] were reviewed, and bibliographies of identified papers were screened.

### Inclusion and exclusion criteria

To be included, instruments had to meet the following criteria:

1. Designed to assess cognitive and physical ability to manage medications;

2. Utilised standardised, structured, quantitative, direct observation of patients' medication management performance;

3. Described in adequate detail to enable test to be replicated (all items and instructions published or able to be obtained from authors);

4. At least one published report of psychometric evaluation of instrument performance.

Instruments were excluded if they relied on self-report or informant-report or included non-medication domains (e.g. financial management, cooking) without providing separate psychometric data on the medication domain. When studies appeared to fulfil the inclusion criteria, or in cases of doubt, full-text copies were obtained. The literature search was conducted by one author (RAE), but before any instrument was excluded it was independently reviewed against the inclusion criteria by the second author.

To ensure that we had all available psychometric data, a search of Medline, EMBASE, IPA and Google was performed using the full and abbreviated name of each instrument (May 2008), and the authors of studies that described development of a novel tool were contacted by email to request unpublished data, tool revisions and instructions for use. Information on each instrument was compiled using a standard template that included: instrument purpose, components of assessment, administration and scoring procedures, time to administer, validation samples, reliability and validity data.

### Methodological quality of validation studies

Validation studies were reviewed independently by both authors and methodological quality was rated using criteria based on the Standards for Reporting of Diagnostic Accuracy [see Additional file 1] [18]. In cases of disagreement studies were discussed and consensus reached.

### Instrument validity

Because there is no accepted 'gold standard' measure of medication management ability against which criterion validity can be judged [8,10], we reviewed content validity, construct validity, and responsiveness to change [19]. Content validity was judged through a consensus process involving both authors, by determining whether instruments assessed each of the main skill-areas necessary for medication management: 1) identify medications (e.g. by reading the label or recognising packaging), 2) access medications from packaging, 3) comprehend and explain or demonstrate medication instructions, 4) recall information, and 5) administer medications [1,20]. Construct validity was judged by reviewing the strength of one or more of the following: association between subjects' performance on the instrument and tests of related constructs (e.g. medication adherence, IADL performance, cognitive function, health literacy), convergent validity (where performance on the instrument was compared with another measure of medication management ability such as a carers' judgement or performance on a second medication management instrument), discriminative or 'extreme groups' validity (where the instrument was administered to a group of subjects known to have problems with medication management or cognitive performance and normal controls), or comparison of instrument performance with subjects' self-reported medication management or place of residence (i.e. independent living versus supported care).

Based on instruments' reliability, validity and quality of validation studies, together with ease and practicality of use (e.g. administration time), we selected instruments that in our opinion showed most potential for use in clinical practice.

## Results

Forty-nine published reports describing development and/or application of 32 instruments were identified. Eighteen did not meet the inclusion criteria (Table 1) [21-38].

**Table 1:** Excluded medication management assessment instruments

**Instrument**	**Author**	**Reason for exclusion**
Assisted Living Facilities Medication Assessment	Meade 2001 [21]	• No psychometric evaluation
Dexterity Test	Begley 1997 [22]	• Insufficient detail* • No psychometric evaluation • Assessment of physical ability only
Everyday Cognitive Battery	Allaire 1999 [23]	• Multi-domain tool† • Insufficient detail* • Assessment of cognitive ability only
Everyday Problems Test	Willis 1996 [24]	• Multi-domain tool† • Insufficient detail* • Assessment of cognitive ability only
Functional Limitations Assessment	Hurd 1986 [25]	• No psychometric evaluation
Medication Management Test	Corrigan 1994 [26]	• Insufficient detail*
Medication Regimen Adherence Capacity Test	Fitten 1995 [27]	• Psychometric evaluation of only part of the test
Observed Tasks of Daily Living (OTDL)	Diehl 1995 [28]	• Multi-domain tool†
Occupational Therapy Assessment Scale (OTAS)	Fairbrother 1997 [29]	• Multi-domain tool† • Insufficient detail*
Occupational Therapy Assessment of Performance and Support (OTAPS)	Nadler 1993 [30]	• Multi-domain tool† • Insufficient detail*
Reading/Comprehension and Task Performance Tool	Bailey 1995 [31]	• Insufficient detail*
Regimen Comprehension Scale	Yamada 2001 [32]	• Assessment of cognitive ability only • Insufficient detail*
Structured Assessment of Independent Living Skills (SAILS)	Mahurin 1991 [33]	• Multi-domain tool†
Self-Administration of Medication tool	Manias 2006 [34]	• No standardised structured observation of task performance
Self-Medication Risk Assessment Instrument	Fuller 2005 [35]	• No standardised structured observation of task performance
Structured Home Interview	Sedgeworth 1990 [36]	• Insufficient detail*
Timed Activities of Daily Living (TIADL)	Owsley 2002 [37]	• Multi-domain tool† • Assessment of cognitive ability only
Virtual Reality Apartment Medication Management Assessment	Kurtz 2007 [38]	• Assessment of cognitive ability only

* insufficient detail to enable tool to be replicated

† multi-domain tool with no separate psychometric evaluation of the medication component

The 14 instruments included in the review fell into two broad categories: those that used the patients' own medications as the basis for assessment [39-41], and those that used a simulated medication regimen [42-52]. Two tools used a simulated regimen plus assessment of knowledge of the patients' own medications [42,44]. A description of the included instruments is provided in Additional file 2[53,54].

### Content validity

The extent to which instruments assessed medication management skills was variable [see Additional file 3]. The theoretical framework or method used to select or develop test items was not reported in most cases. All instruments apart from MedMaIDE [41] required the patient to identify one or more medication either by reading a label or some other method. All instruments required the patient to open at least one type of medication packaging, and all except MAI [42] and MMPT [52] required the patient to remove a dose. All instruments except MMPT [52] required the patient to comprehend and either explain or set-out at least one medication. Ability to administer medication (e.g. swallow an oral dose) was assessed by two tools (MedMaIDE, PA) [41,44]. Six instruments assessed ability to recall information [40-42,44,46,49].

### Administration time

Most instruments were reported to take between 5 and 15 minutes to administer [see Additional file 2]. Instruments that included an assessment of medication knowledge in addition to the medication-taking task required more time (15–30 minutes) [40-42,44,47]. Administration time may be longer in patients with cognitive impairment, and more variable for instruments that use patients' own medications because of the variable number of medications [55]. Five instruments had a time limit or timed component [42,45,49-51].

### Quality of validation studies

The quality of validation studies was generally low to moderate [see Additional file 4]. Most instruments were tested in relatively small, homogenous samples recruited from a single setting. Some studies specifically excluded cognitively impaired individuals [42,45,49,50,55], and only one instrument was tested in a representative sample of older people [52]. For 10 instruments the person administering the assessment was not blinded to results of the reference standard and other validation tests (e.g. cognitive function, IADL scores), or this information was not provided [40-45,47,50-52]. In other cases, the rater was blinded to some, but usually not all, patient data [39,46,48,49,56]. Most studies did not adequately describe reasons for patient exclusion or their characteristics relative to the study subjects [40-43,45-49,51], or whether study subjects were currently managing their own medications [42-44,46-51], making generalisation of findings difficult and introducing potential spectrum bias [13,18,57]. In six studies the reference standard was not applied to all subjects [9,44,45,47-49]. Only one validation study (MedMaIDE) reported sensitivity and specificity with respect to its ability to predict a medication-related outcome [41].

Only three instruments were evaluated in multiple populations by more than one research group (MMEI, DRUGS, MMAA) [see Additional file 4]. The MMAA has been most widely used, but all except one study involved subjects with schizophrenia or other mental health disorders, limiting generalisability.

In many cases, inadequate information was provided to allow the test to be replicated, so contact with the developers was necessary. Some instrument developers have made modifications after publishing the instrument, seemingly without further validation (e.g. MMAA, MMT-R).

### Reliability

Few instruments were subjected to reliability testing [see Additional file 4]. Only three (DRUGS, MedMaIDE, MMT) have inter-rater reliability data available [39,41,47], and for two of these (DRUGS, MMT) the methods by which the data was obtained was not adequately described [39,47]. Three instruments have test-retest reliability data (DRUGS, MedMaIDE, MMAA) [39,41,49]. Internal consistency was reported for four instruments (HMS, MedMaIDE, MMT, MM Test) [41,46,47,50]. No instrument has had its reliability confirmed independently by a second research group.

### Construct validity

#### Cognitive function

The association between medication management performance and measures of cognitive function was investigated for 11 instruments, and an association was found with 10 of these [see Additional file 4]. The association in most cases was moderate (correlation coefficient 0.4–0.6) (DRUGS, MedTake, MedMaIDE, MMAA, MAT) [39-41,49,51,58-60]. For one instrument (MM Test), which was originally developed as a screening tool for dementia, the association was stronger (r = 0.66, p < 0.001) [46]. For four instruments no correlation coefficient was provided (MMEI, MMT, MMT-R, HMS) [43,47,48,50,61-63]. Lack of association with SM Task may have been due to the small homogenous sample (n = 20, MMSE >25/30) [45].

#### Health literacy

The association between medication management performance and validated measures of health literacy was reported for two instruments [see Additional file 4]. MedTake scores were associated with performance on the Rapid Estimate of Adult Literacy in Medicine (REALM) (r = 0.344; p < 0.01) [64]. In multiple regression analysis, REALM was the strongest predictor of MedTake score, accounting for 27% of variance. In another study, DRUGS scores increased with increasing literacy (p = 0.001) [65].

#### Activities of daily living

Self-reported basic ADL performance was included in validation studies for three instruments [see Additional file 4] [39,41,61], and a positive association with medication management was found in only one (MMEI) [61]. This is not surprising since medication management is a high-level ADL and participants in most studies were relatively high functioning. In the study where an association was reported, 46% of subjects were dependent for at least one basic ADL [61].

Self-reported IADL performance was assessed in studies with five instruments, and an association was reported with three of these (HMS, MAT, MMT-R) [see Additional file 4] [48,50,51]. There was no association between DRUGS scores and self-reported IADL performance [39], while in the MedMaIDE validation study IADL data was collected but not reported [41]. Given the limitations of self-reported functioning, observed IADL performance would be a more valid comparator [6,8]. This was available for two instruments, and a moderate association was found in both cases. The MMAA instrument was associated with scores on the Direct Assessment of Functional Status instrument, a multi-domain functional performance measure (r = 0.51, p < 0.001) [49]. The MAT instrument was associated with performance on the food preparation item of the Naturalistic Action Test (r = 0.46, p < 0.001) [51].

#### Medication adherence

Self-reported adherence was assessed in studies with four instruments [see Additional file 4]. Although there was an association with two items on the MAI, overall test performance was not reported [42]. There was no association with instrument performance in a study using DRUGS and MMAA [55], and another using MMT [47]. There was an association between MMAA total-score and self-reported adherence (r = 0.37, p < 0.01) in a subgroup analysis of independent-living older patients with mental illness, but validity of this association is questionable because there was no association with pill count adherence in the same study [66].

Objective measures (e.g. pill count, medication refills) may provide a more accurate estimate of medication adherence, and these were included in studies with five instruments [see Additional file 4]. The primary validation study for the MMAA included a subgroup analysis of 22 subjects with schizophrenia for whom 12 months of medication refill data was available, and reported 67% agreement (p < 0.001) in classification of patients into adherent versus nonadherent using medication refill and MMAA pill-count score (not MMAA total score) [49]. However >50% of subjects resided in supported care and may not have been responsible for managing their own medications. In another study, there was no association between MMAA total score and pill count adherence in older patients with mental illness, but in a post-hoc subgroup analysis of subjects who lived independently there was a weak association with the MMAA pills-under score (r = 0.3, p < 0.05) [66]. There was a moderate association between 30-day pill count adherence and MedMaIDE scores (r = -0.52, p-value not reported) [41]; in a regression analysis, 27% of the variance in pill count adherence was accounted for by the MedMaIDE score (p < 0.001), and using a score of >1 as the cut-off, sensitivity was 68% and specificity 83% [41]. No association was found between adherence and instrument scores in studies using the MMEI, SM Task and PA [45,62,67], but in two studies adherence was assessed for only 16 and 14 subjects respectively [45,62], while interpretation of the third study is limited by poor description of the methodology [67].

#### Other measures of medication management ability

The PA instrument was compared with success in an inpatient self-medication program or self-medication at home. Although the authors reported 69% predictive accuracy, neither the cut-score for the instrument nor the criteria for successful self-medication was reported [44]. Also, treating physicians' and nurses' opinions had similar predictive accuracy without using the instrument (62% and 58% respectively) [44]. There was a moderate correlation between MMAA and DRUGS scores in 52 independently living patients (r = 0.56, p = 0.000) [55].

#### Discriminative (extreme groups) validity

In a study where MMAA performance in older schizophrenic patients and normal controls was compared, patients scored significantly lower than controls [49]. When MMT-R performance in HIV-positive subjects with and without cognitive impairment was compared, those with impaired cognition were more likely to fail [48]. Similarly, when Fulmer et al split their sample of older people into normal cognitive status and cognitively impaired, poor scores on the MM Test were obtained significantly more often in subjects with cognitive impairment [9]. In cross-sectional studies both DRUGS and MAT scores were significantly lower in assisted living subjects compared with independent living subjects [39,51], although it is possible that this finding is confounded by lack of medication familiarity and recent practice among assisted living subjects.

#### Independent medication management

The relationship between medication management task performance and whether or not patients were independently managing their medications at home was reported for four instruments (DRUGS, MMEI, MMTest, MMPT) [39,43,46,52]. Although performance was related to self-reported or caregiver-reported medication management in all four studies, again this could be confounded by lack of medication familiarity or practice for those not managing their own medications.

#### Place of residence

In a prospective study, baseline DRUGS scores predicted residence in assisted living at 6 months but not 12 months [56]. Change in DRUGS scores between baseline and 12 months was associated with residence in assisted living at 12 months [56].

### Responsiveness to change

Only two instruments were evaluated in longitudinal studies. The DRUGS instrument demonstrated responsiveness to changes in cognitive function over 12 months in 52 highly functioning older people [56], and over six months in an interim analysis of an unpublished study with a more diverse group of 58 older people [68]. MMAA scores increased following a 12 week group medication skills training program in an uncontrolled pilot study involving 16 patients with bipolar disorder [69].

### Head-to-head comparisons

Only one study has been published in which two medication management instruments (MMAA and DRUGS) were administered to the same population [55]. As noted above, there was an association between the two instruments' scores, suggesting they were measuring the same construct. Patients performed better on DRUGS than MMAA, with 87% and 18% attaining the maximum possible score, and mean scores of 92% and 78% respectively [55]. Better performance on the DRUGS instrument may be partially explained by medication familiarity since DRUGS uses the patients' own medications. It may also be a result of the different scoring systems. Six patients (11%) refused to take the DRUGS test, usually because they did not want to bring their medications to the clinic. In contrast only three patients refused to take the MMAA test: two felt they would be unable to do it (both had MMSE 20/30) and one cited 'lack of time' [55].

## Discussion

There have been many attempts to develop a standardised, objective, quantitative measure of patients' ability to manage their own medications, but no published instrument currently has sufficient evidence of reliability and validity to allow it to be recommended for routine use in clinical practice to predict outcomes such as medication adherence or errors. Most instruments have had limited evaluation of reliability, and although most have demonstrated an association with at least one related construct there is limited evidence of ability to predict successful independent medication management in the real world. A wide variety of reference standards and comparators has been used for validity assessment, making it difficult to compare instruments. All validation studies had methodological shortcomings and most were conducted in small homogenous populations, which can lead to overestimation of instrument validity [13,70]. Therefore the data for most instruments needs to be regarded as preliminary, until confirmed in larger, well-designed studies. The possibility of publication bias leading to over-estimation of instrument validity also needs to be considered.

Based on current evidence, the instruments that in our opinion show most promise and may warrant further investigation are: DRUGS, MAT, MedMaIDE, MMAA, MM Test and MMEI.

DRUGS [39] and MedMaIDE [41], which both use patients' own medications, are the only instruments with evidence of both inter-rater and test-retest reliability, although this needs to be confirmed by researchers other than the instruments' developers. The MedMaIDE instrument demonstrated construct validity through moderate correlations with pill-count medication adherence and cognitive function, albeit in a single small study. A disadvantage is that it requires 30 minutes for administration. The DRUGS instrument demonstrated construct validity through its associations with cognitive function, health literacy, independent medication management and MMAA performance. It has also been shown to be responsive to change, and has the advantage that it has been validated in several patient groups by three different research groups. It requires up to 15 minutes for administration.

The MAT [51] and MMAA [49] both require patients to set-out 24 hours of a simulated medication regimen. They take around 15 minutes to administer, but the MMAA requires an additional 45–60 minute delay between explaining the regimen to the patient and task completion. Validity of both instruments is supported by moderate correlations with cognitive function and observed IADL performance. MAT performance has also been associated with level of supported care, and MMAA performance has been associated with DRUGS scores. MAT has no reliability data, and has only been validated in one small study. A strength of the MMAA is that it has been validated by multiple research groups in large numbers of patients, although mostly patients with mental health disorders. Despite its widespread use, it has never been subjected to inter-rater reliability testing. MAT uses more medications but has a simpler scoring method that incorporates task completion time, which may improve discriminative validity [11].

MM Test [46] and MMEI [43] also use simulated medications but have the advantage of being faster to administer, requiring approximately 5 minutes. MM Test is strongly associated with cognitive function and moderately associated with independent medication management. It has good internal consistency, but inter-rater and test-retest reliability is unknown. MMEI has shown weaker association with these two variables but has been evaluated by three research groups. It has not been subjected to reliability testing.

Although further research is needed before any of these instruments could be used to reliably predict medication outcomes, they could be used to qualitatively identify barriers to successful medication management. Used in this way they could assist health professionals to identify individual patients' need for education, regimen simplification, medication assistance or medication aids [44]. In this context, the DRUGS, MedMaIDE, MedTake, MAI, MMEI and PA instruments may provide the most information about physical barriers and medication knowledge, while the MM Test, MMAA and MAT instruments may provide the best assessment of medication-related cognitive abilities. A limitation of most instruments in the assessment of physical barriers is that they assess ability to access medications from vials only, whilst increasingly medications are supplied in foil or blister-packs which can also be difficult for patients to manage [1], and no instrument assesses ability to self-administer complex dose-forms such as inhalers and eye drops.

Our choice of instruments warranting further study differs from recommendations made in an earlier review by Farris and Phillips [12]. Consistent with our recommendations they selected DRUGS, MedMaIDE and MMAA, however they also selected MMPT and HMS. We did not recommend MMPT because, although it is a brief test, it assesses a limited range of medication management skills and it has limited construct validity data and no reliability data.

The HMS also has limited construct validity and reliability data and seems to offer no advantage over similar, better-validated simulated medication regimen instruments such as MMAA or MAT.

Although the choice of instruments is somewhat subjective, a strength of our review compared with that of Farris and Phillips is that methodological quality of validation studies was formally assessed and, together with psychometric data, considered in the selection process. We also considered instruments' applicability in all settings, not just primary care. A limitation of both reviews is that searches were limited to English language publications, possibly leading to some instruments being overlooked.

Farris and Phillips suggested that instruments using patient's own medications may be preferable due to greater test authenticity and ability to assess patients' medication knowledge [12]. These instruments also have the advantage of not requiring special materials or props. However they also have potential disadvantages: patients' own medications need to be available, which may be problematic in inpatient and clinic settings, and patients may be reluctant to provide their medications for the test [55]. These instruments may be best suited to situations where assessment occurs in patients' homes or as part of a 'brown bag medication review'. Instruments using simulated medications provide a higher level of test standardisation, allowing comparisons to be made between patients and within patients over time. They may be suited to settings where patients' own medications are unavailable or medications are being newly commenced, such as clinics or hospital wards. Because these tests use a medication regimen that is not familiar to the patient, they are able to assess capacity to manage new medications (e.g. after discharge from hospital). To avoid bias due to medication familiarity, it may be preferable that simulated regimens contain mock medications (e.g. as in MMAA), rather than real medications.

No study has reported on the acceptability of medication management assessment instruments to patients. Administration of these instruments in clinical practice could potentially be stressful for frail elderly patients, especially if they believe that poor performance may lead to loss of independence (e.g. placement in residential care). It is possible that instruments using patients' own medications may be less threatening compared to unfamiliar simulated regimens, but this has not been studied and is not supported by a finding that fewer patients declined to take the MMAA test than the DRUGS test in the only study that has compared two instruments [55].

Instrument development is ongoing. We are aware of three additional instruments that were not included in this review, all using simulated medications. Two had only been published in abstract or thesis form [71,72], while the third was published after this review was completed and assessed cognitive medication management abilities only [73]. There is a need for further research to confirm the reliability and validity of published standardised medication management assessment instruments. The quality of validation studies needs to be improved, for example by blinding testers to results of reference standard assessments. When medication adherence is used for instrument validation, objective adherence measures should be used, and only patients who are responsible for managing their own medications included. However, it may be unrealistic to expect any of the current instruments to predict medication adherence with a high level of accuracy. Since most instruments assess only the cognitive and physical abilities required to manage medications, their results reflect subjects' capacity to accomplish the tasks, and performance in real life may not fulfil the potential indicated by capacity [6,10,46]. A range of additional factors contribute to whether or not a patient will adhere to a medication regimen, including: motivation, belief that the therapy is necessary and effective, self-efficacy, relationship with healthcare providers, lifestyle and cultural factors and financial capacity [8,10,20]. The MedMaIDE instrument, which had a moderate association with pill-count adherence included items related to accessing ongoing medication supplies, in addition to physical and cognitive abilities [41]. Inclusion of items addressing additional adherence-related factors could potentially increase predictive accuracy. Novel validation methods may be required in future studies, due to the lack of a 'gold standard' for medication management ability. For example, the instrument could be administered to hospital patients entering an inpatient self-administration of medication program, allowing performance on the instrument to be compared with performance on the program.

## Conclusion

A number of performance-based instruments exist to assess older patients' ability to manage their own medications. These instruments may be useful for identifying physical and cognitive barriers to successful medication management, but further studies are needed to determine whether they are able to reliably predict medication outcomes such as adherence or errors.

## Abbreviations

DRUGS: Drug Regimen Unassisted Grading Scale, HMS: Hopkins Medication Schedule, MAI: Medication Assessment Instrument, MAT: Medication Administration Test, MedMaIDE: Medication Management Instrument for Deficiencies in the Elderly, MMAA: Medication Management Ability Assessment, MMEI: Medication Management Evaluation Instrument, MMPT: Medication management performance tests, MMT: Albert's Medication Management Test, MMT-R: Albert's Medication Management Test-Revised, MM Test: Gurland's Medication Management Test, PA: Pharmacy Assessment, SM Task: Self-Medication Task.

## Competing interests

The authors declare that they have no competing interests.

## Authors' contributions

RAE conceived and designed the study, conducted the literature search, data extraction and analysis, and prepared the manuscript. JLM contributed to study design and data analysis, and reviewed drafts of the manuscript.

## Authors' information

RAE is a senior clinical pharmacist at Austin Health and clinical senior lecturer in aged care pharmacy practice at Monash University. JLM is a senior lecturer in clinical pharmacy at Monash University.

## Pre-publication history

The pre-publication history for this paper can be accessed here:



## Supplementary Material

Additional file 1 **Supplemental table S1**. Criteria used to assess methodological quality of validation studies. Click here for file

Additional file 2 **Supplemental table S2**. Description of medication management assessment instruments included in the review. Click here for file

Additional file 3 **Supplemental table S3**. Medication management skills assessed by included instruments. Click here for file

Additional file 4 **Supplemental table S4**. Reliability and validity of medication management assessment instruments. Click here for file
