# Supplementary material for: Standardised assessment of patients' capacity to manage medications: a systematic review of published instruments
Source: BMC Geriatr. 2009 Jul 13;9:27. doi: 10.1186/1471-2318-9-27 (PMC2719637; doi:10.1186/1471-2318-9-27)
Supplement: Additional file 1 — Supplemental table S1. Criteria used to assess methodological quality of validation studies. [file 1471-2318-9-27-S1.doc]

## Supplemental table 1. Criteria used to assess methodological quality of validation studies.

|  | **Item** | **Criteria** |
| --- | --- | --- |
| 1 | Sample size | 2 if ≥ 100 subjects  1 if 50-99 subjects  0 if < 50 and not corrected for multiple testing |
| 2 | Study design | 2 if prospective validation study  1 if cross-sectional validation study  0 if retrospective validation study |
| 3 | Subject selection  (selection bias) | 2 if subjects selected randomly or consecutively  1 if selected in a non-random, non-consecutive way other than described below (e.g. convenience sample)  0 if subjects selected on the basis of having a known problem with medication management or functional performance (or information not provided) |
| 4 | Excluded subjects | 2 if number of subjects who met inclusion criteria but did not complete the study, and reasons, are described  1 if above criteria partially met  0 if above criteria not met |
| 5 | Subject characteristics (spectrum bias; generalisation) | 2 if study setting and population (age, gender, education, cognitive function, medication management status) described  1 if above criteria partially met  0 if above criteria not met |
| 6 | Test description | 2 if methods for performing and scoring the index test described in sufficient detail  1 if methods for performing and scoring the index test not described but able to be obtained from author  0 if methods for performing and scoring the index test not described and unable to be obtained from author |
| 7 | Test administration | 2 if number, training and expertise of the persons executing the index test and reference standard/validation tests described  1 if above criteria partially met  0 if above criteria not met |
| 8 | Reliability testing | 2 if methods for determining reliability adequately described  1 if above criteria partially met  0 if above criteria not met |
|  | **Item** | **Criteria** |
| 9 | Reference standard/ validation test(s) | 2 if reference standard/validity test(s) and rationale are described  1 if above criteria partially met  0 if above criteria not met |
| 10 | Validation testing (verification bias) | 2 if reference standard/validation tests applied to every subject  1 if reference standard/validation tests applied to most, but not all, subjects  0 if reference standard/validation tests applied only to subjects who performed poorly in medication management test (or information not provided) |
| 11 | Time interval | 2 if time interval from index test to the reference standard/validation tests, and any treatment administered between them, is described.  1 if above criteria partially met  0 if above criteria not met |
| 12 | Blinding (review bias) | 2 if index test conducted independently of all reference standards/validity tests (with blinding of raters)  1 if above criteria partially met  0 if above criteria not met |
| 13 | Units & cut-points | 2 if definition and rationale for the units, cutoffs, and/or categories of the results of the index test and the reference standard described  1 if above criteria partially met  0 if above criteria not met |
| 14 | Test results | 2 if a cross tabulation or distribution of the results of the index test by the results of the reference standard provided  1 if above criteria partially met  0 if above criteria not met |
| 15 | Predictive accuracy | 2 if estimates of predictive accuracy and measures of statistical uncertainty reported (e.g. sensitivity and specificity and/or likelihood ratios, confidence intervals)  1 if above criteria partially met  0 if above criteria not met |
| **Total score** (out of 30) | | |
