# Supplementary material for: Standardised assessment of patients' capacity to manage medications: a systematic review of published instruments
Source: BMC Geriatr. 2009 Jul 13;9:27. doi: 10.1186/1471-2318-9-27 (PMC2719637; doi:10.1186/1471-2318-9-27)
Supplement: Additional file 2 — Supplemental table S2. Description of medication management assessment instruments included in the review. [file 1471-2318-9-27-S2.doc]

## Supplementary table 2. Description of medication management assessment instruments included in the review.

| **Instrument** | **Purpose*** | Description | **Scoring** | **Time (minutes)** | **Supporting studies** |
| --- | --- | --- | --- | --- | --- |
| Instruments that use patients’ own medications | | | | | |
| DRUGS (Edelberg, et al 1999) [39] | To assess patients’ ability to take their own medications independently. Designed to be used in clinic settings as part of a ‘brown bag’ medication review. | Subject performs the following four tasks with each of their medications: 1) identify the medication, 2) open the container, 3) select the correct dose, and 4) report the appropriate timing of doses using a grid marked with specific times of the day as a role-play of their typical day. | Out of 100. Each drug is scored out of 4, with 1 for each correct response/action. This is then converted to a percentage score. The composite score is the overall average score. | 5-15 | Edelberg, et al 2000 [56]  Edelberg, et al 2002 [68]  Hutchison, et al 2006 [55]  Kripalani, et al 2006 [65] |
| MedTake (Raehl , et al 2002) [40] | To assess patients’ ability to correctly take their own oral prescription medications. Designed to be used in clinic settings as part of a ‘brown bag’ medication review. | Subject performs the following four tasks with each of their medications: 1): open the container and simulate taking the first dose of the day, 2) describe indication, 3) describe food or water co-ingestion, 4) describe regimen (i.e. number of times per day). | Out of 100. Each drug is scored out of 4, with 1 for each correct response (dose, indication, food/water co-ingestion, regimen). This is then converted to a percentage score. The composite score is the overall average score. | 30-45† | Raehl, et al 2006 [64] |
| MedMaIDE (Orwig, et al 2006) [41] | To identify deficiencies in older adults’ ability to self-medicate at home.  Designed to be used in patients’ homes. | 20 items covering three areas: 1) Medication knowledge (name all medications, indications and dose regimens); 2) Medication-taking ability (access doses from packaging, fill glass of water, sip water); 3) Access to ongoing supply of medication (awareness of refills/repeats, able to arrange supply). | Out of 13 (only 13 of the 20 items are scored). A yes (or able) response scores 0, and a no (unable) response scores 1. Therefore the higher the score the less capable the patient is. | 30† | Nil |
| Instruments that use simulated medication tasks | | | | | |
| MAI (Murray, et al 1986) [42] | To assess patients’ medication knowledge and skills and identify barriers to appropriate medication management. | Two components: Part A (Skills component) requires the subject to perform the following tasks: 1) open 3 types of medication vial, 2) read a standard medication label and auxiliary warning label, 3) distinguish tablet colours. Part B (Knowledge component) tests the subject’s knowledge of their own medications (from memory, without looking at medication labels): 1) reason for use, 2) duration, and 3) how many dosage units are taken throughout the day. | No scoring system described in original study. In a later study (same research group), items were scored as 0 (incorrect or unable), 1 (partially correct or able with difficulty) and 2 (correct or able) [72]. | 15-30§ | Hope, et al 2004 [53] |
| **Instrument** | **Purpose*** | Description | **Scoring** | **Time (minutes)** | **Supporting studies** |
| Instruments that use simulated medication tasks *(continued)* | | | | | |
| MMEI (Meyer, et al 1989) [43] | To assess patients’ functional ability to take medication. | Subject performs the following tasks: 1) read a prescription label attached to a standard medication vial; 2) open and close a child-resistant cap; 3) open and close a non-child-resistant cap; 4) remove two tablets from an opened vial; 5) describe a “three times a day” dosing regimen; 6) differentiate tablets by colour. | No scoring system described in original study. Ruscin, et al subsequently introduced a scoring system out of 5. (Items 1, 2/3, 4, 5 and 6 each scored as 1 for “able” and 0 for “unable”) [59]. | 5 | Ruscin, et al 1996 [61]  Fritsch, et al 1998 [62] |
| PA‡  (Romonko & Pereles 1992) [44] | To identify deficiencies in medication self-administration ability (to guide hospital discharge-planning decisions). | A 28-item instrument, divided in three sections:   1. Mental status and functional ability  (e.g. ability to read a label, open vials, remove tablets from a vial, differentiate tablets by colour, manipulate a multi-compartment medication aid, describe a three times daily regimen, hear instructions & swallow pills) 2. Daily routine at home  (e.g. regular meals, system for taking medications when away from home) 3. Medication and dosing considerations  (e.g. medication knowledge, storage/re-packing of medicines, regular pharmacy, adherence aids, etc.) | No scoring system described. Response/performance for each item is rated as yes or no, so if scored as 1 or 0 a total score out of 28 could be obtained. | 20 | Johnson, et al 2005 [67] |
| SM Task (Isaac, et al 1993) [45] | To assess patients’ medication planning ability (ability to successfully administer a new medication) | The subject is asked to read out loud the labels of three dispensed medications, open each bottle, and plan one week’s administration by placing the appropriate amount of each drug in a Dosett. One tablet needs to be split in halves and another into quarters. | Six scores are generated: 1) reading accuracy, 2) bottle-opening speed, 3) bottle opening success, 4) pill cutting success, 5) errors in medication timing, 6) errors in dosing. | Max. 20¶ | Nil |
| **Instrument** | **Purpose*** | Description | **Scoring** | **Time (minutes)** | **Supporting studies** |
| Instruments that use simulated medication tasks *(continued)* | | | | | |
| MM Test (Gurland, et al 1994) [46] | To measure high-level adaptive cognitive functioning in early dementia [46], and assess real-life capacity to manage medications [9]. | A box containing six medication vials is placed facing the subject, who is instructed to demonstrate the following: (1) learn and immediately recall the number of pills in one vial; (2) subtract three pills from nine; (3) calculate how long the pills would last on a regimen of 3/day; (4) work out the days for taking pills on an alternate day dosing regimen over two weeks; (5) pick out a medication vial based on its description; (6) remove 3 pills then replace them and return the vial; (7) select the same vial again and remove 3 pills on a delayed signal; (8) describe the distinguishing features of a medication vial designated by its therapeutic use that was explained earlier. | 17 items, each separately scored; summated score ranging from 0 to 46, where 0 = perfect performance. | 5 | Fulmer, et al 1997 [9] |
| MMT (Albert, et al 1999) [47] | To predict patients’ ability to adhere to anti-retroviral medication regimens. | Two components: A. “Pill dispensing“; and B. “Medication inference”.  In Part A, five plastic pill bottles with dried beans are labelled with directions. One is labelled to be used PRN. Subjects are required to transfer the right number of pills to a compartmentalised DAA. They must recognise that the PRN medication should not be placed in the DAA. In Part B, the subject is required to answer 15 questions about the medications and information from an over-the-counter medication insert. | In Part A, subjects are scored on the percentage of medications correctly placed in the DAA, ranging from 0 (all five wrong) to 100% (all correct). In Part B items are scored dichotomously and the total is expressed as percent correct. | 15-25 | Albert, et al 2003 [63] |
| MMT-R (Heaton, et al 2004) [48] | To assess HIV-positive patients’ functional medication management ability. | MMT-R retains the two parts described above, but part A utlises 3 medications instead of 5, and part B includes 7 items instead of 15.  Note: MMT-R forms part of the Functional Deficiency Score (FDS), a multi-domain functional assessment instrument. | Scored out of 10. | 10 | Nil |
| **Instrument** | **Purpose*** | Description | **Scoring** | **Time (minutes)** | **Supporting studies** |
| Instruments that use simulated medication tasks *(continued)* | | | | | |
| MMAA (Patterson, et al 2002) [49] | To assess ability of older mental health patients to independently manage medications. | Four plastic pill bottles with dried beans are labelled with directions. The interviewer describes the medication regimen to the subject, then 45-60 minutes later the subject is asked to walk through their day, saying when they would wake up, eat meals, and take each medication. They are required to dispense the pills for each dose and hand them to the interviewer. | Four scores reported:  A) Total MMAA score (out of 25): One point deducted for each deviation from prescribed regimen (i.e. too many, too few pills, or incorrect timing in relation to food).  B) Total number of pills correct (out of 21)  C) Number over: Total number of pills exceeding prescribed number  D) Number under: Total number of pills fewer than prescribed number  Note: Since publication the scoring system has been revised and total MMA score is now out of 33 (personal communication, B Kelly 13/5/08). | 15# | Jeste, et al 2003 [60]  Hutchison, et al 2006 [55]  Pratt, et al 2006 [66]  Depp, et al 2007 [69]  Kurtz, et al 2007 [38]  Heinrichs, et al 2008 [58]  Depp, et al 2008 [59] |
| HMS (Carlson, et al 2005) [50] | To provide an objective measure of older adults’ ability to understand and implement a medication regimen. | Two parts: 1) ‘Schedule’ and 2) ‘Pillbox’.  Part 1: the subject is given a hypothetical scenario in which she has been prescribed medications to treat an infection (antibiotic: 1 tds 30ac; aspirin 2 q4h with food; water: 1 glass q2h). The subject is asked to plan a schedule for taking the medications and water during the course of a day using a paper-based schedule with hours of the day marked. Max. 8 minutes allowed.  Part 2: Subject is given labelled, non-child-resistant vials containing aspirin and antibiotic placebo pills and a four-compartment pillbox (labelled: morning, lunch, dinner and bed), and instructed to set out 1 day of the tablets into the appropriate compartments. Max. 4 minutes allowed. | Maximum score = 11 (Schedule, out of 9; Pillbox, out of 2). The schedule is scored for compliance with dose-timing, method, daily dosage of medications and proper intake of food and water. The pillbox is scored for placement of 1 antibiotic capsule in three of the four compartments and two aspirin tablets in all four compartments. Two methods to adjust scores for completion time were described.  In a subsequent study from the same researchers, Part 2 (pillbox) was scored with a 3-point system (1 = both medications incorrectly placed, 2 = 1 medication correctly placed, 3 = both medication correctly placed). Scores were time adjusted [73]. | 5-15 | Windham, et al 2005 [54] |
| **Instrument** | **Purpose*** | Description | **Scoring** | **Time (minutes)** | **Supporting studies** |
| Instruments that use simulated medication tasks *(continued)* | | | | | |
| MAT (Schmidt, et al 2005) [51] | To assess patients’ functional and cognitive ability to self-administer prescription medications. | Four boxes, labeled ‘morning’, ‘noon’, ‘evening’ and ‘bedtime’ are placed in front of the subject. Ten labeled medication vials (containing placebo pills representing ten commonly used medications) are provided. Subjects are asked to read the administration directions from the bottles and place the correct number of pills into the appropriate box. | Single summated score from 0 to 100. Scoring includes task completion time and number of errors (omission, commission, sequence). To score 100 subject needs to complete task within 5 minutes with no errors; points (in multiples of 10) are lost for slower completion (max. 15 minutes) and errors; a zero score indicates 8 or more errors. | 5-15 | nil |
| MMPT‡ (Beckman, et al 2005) [52] | To identify visual, physical and cognitive limitations that can effect older persons’ medication management. | Five tests: 1) read instructions on a standard medication vial, 2) open a standard vial, 3) read and interpret maximum daily dose instructions on a box of aspirin tablets, 4) calculate how many days a supply of pills will last based on dosage and number of pills in the box, 5) calculate amount a change from a 100-crown note when purchasing two medications for a total of 64 crowns. | No scoring system described, but study results suggest a scoring system out of 5 was used (1 point for each ‘test’, where 0 = correct and 1 = incorrect) | Not reported | Nil |

DRUGS = Drug Regimen Unassisted Grading Scale; HMS = Hopkins Medication Schedule, MAI = Medication Assessment Instrument; MAT = Medication Administration Test; MedMaIDE = Medication Management Instrument for Deficiencies in the Elderly; MMAA = Medication Management Ability Assessment; MMEI = Medication Management Evaluation Instrument; MMPT‡ = Medication management performance tests; MMT = Albert’s Medication Management Test; MMT-R = Albert’s Medication Management Test-Revised; MM Test = Gurland’s Medication Management Test, PA‡ = Pharmacy Assessment; SM Task = Self-Medication Task.

* Purpose for which tool was developed, as described by tools’ developers

† Includes time to record additional data that is not part of the scored assessment

‡ Abbreviation assigned by authors of this manuscript

§ Varies depending on participant's cognitive status and interviewer's experience (Personal communication, M Murray 7/11/07)

¶ Maximum 20 minutes allowed for test completion (Personal communication, R Tamblyn, 15/8/07)

# Plus additional 45-60 minute delay between instruction and task completion.
