# Supplementary material for: Standardised assessment of patients' capacity to manage medications: a systematic review of published instruments
Source: BMC Geriatr. 2009 Jul 13;9:27. doi: 10.1186/1471-2318-9-27 (PMC2719637; doi:10.1186/1471-2318-9-27)
Supplement: Additional file 3 — Supplemental table S3. Medication management skills assessed by included instruments. [file 1471-2318-9-27-S3.doc]

## Supplementary table 3. Medication management skills assessed by included instruments.

| **Instrument** Medication management skills assessed | **DRUGS** [39] | **Med-Take** [40] | **Med-MaIDE** [41] | **MAI** [42] | **MMEI** [43] | **PA** [44] | **SM Task** [45] | **MM Test** [46] | **MMT** [47] | **MMT-R** [48] | **MMAA** [49] | **HMS** [50] | **MAT** [51] | **MMPT** [52] |
| --- | --- | --- | --- | --- | --- | --- | --- | --- | --- | --- | --- | --- | --- | --- |
| Identify medication by any means | X | X† | - | - | - | - | - | X | - | - | X | - | X | - |
| Differentiate tablets by colour | - | - | - | X | X | X | - | - | - | - | - | - | - | - |
| Read standard label | - | - | - | X | X | X | X | - | X | X | - | X | - | X |
| Read ancillary label | - | - | - | X | - | - | - | - | - | - | - | - | - | - |
| Hear instructions | - | - | - | - | - | X | - | - | - | - | - | - | - | - |
| Open standard screw-top vial | X* | X*† | X* | - | X | X | - | X | X† | X† | X† | X† | X† | - |
| Open flip-top vial | X* | X*† | X* | X | - | - | X | - | - | - | - | - | - | X |
| Open child-resistant vial | X* | X*† | X* | X | X | X | X | - | - | - | - | - | - | - |
| Remove dose from vial | X* | X* | X* | - | X | X | X† | X | X | X | X | X^$^ | X | - |
| Remove dose from foil or blister pack | X* | X* | X* | - | - | - | - | - | - | - | - | - | - | - |
| Remove dose from pill-box | - | X* | X* | - | - | X | - | - | - | - | - | - | - | - |
| Split pill | - | - | X* | - | - | - | X | - | - | - | - | - | - | - |
| Pour glass of water | - | - | X | - | - | - | - | - | - | - | - | - | - | - |
| Swallow water or pills | - | - | X | - | - | X | - | - | - | - | - | - | - | - |
| Measure dose of liquid medication | - | - | X* | - | - | - | - | - | - | - | - | - | - | - |
| Prepare injection (e.g insulin) | - | - | X* | - | - | - | - | - | - | - | - | - |  | - |
| Comprehend dose-regimen instructions (e.g. read & explain directions on a label) | - | - | - | - | X | X | - | X | - | - | - | - | - | - |
| Comprehend additional instructions (e.g. read & explain special instructions such as co-ingestion with water/food) | - | - | - | - | - | - | - | - | X | X | X | X | - | - |
| Schedule 24 hours of a medication regimen using a paper-based schedule | X | - | - | - | - | - | - | - | - | - | - | X | - | - |
| Set out medications for one dose-time | X | X | - | - | - | - | - | - | - | - | - | - | - | - |
| Set out 24 hours of medication‡ | - | - | - | - | - | - | - | - | X | X | X | X | X | - |
| Set out 7 days of medication | - | - | - | - | - | - | X | - | - | - | - | - | - | - |

| **Instrument** Medication management skills assessed | **DRUGS** [39] | **Med-Take** [40] | **Med-MaIDE** [41] | **MAI** [42] | **MMEI** [43] | **PA** [44] | **SM Task** [45] | **MM Test** [46] | **MMT** [47] | **MMT-R** [48] | **MMAA** [49] | **HMS** [50] | **MAT** [51] | **MMPT** [52] |
| --- | --- | --- | --- | --- | --- | --- | --- | --- | --- | --- | --- | --- | --- | --- |
| List/name all current (own) medications | - | - | X | - | - | X | - | - | - | - | - | - | - | - |
| Describe indications and/or dosages of own medications | - | X | X | X | - | X | - | - | - | - | - | - | - | - |
| Memory / Recall of information | - | X | X | X | - | X | - | X | - | - | X | - | - | - |
| Perform calculations§ | - | - | - | - | - | - | - | X | X | X | - | - | - | X |
| Judgement & consequences (e.g. knows what to do if a dose is missed) | - | - | - | - | - | - | - | - | X | X | - | - | - | - |
| Motivation/insight (belief that their medications are necessary) | - | - | - | X† | - | - | - | - | - | - | - | - | - | - |
| Read & interpret information in package insert or on OTC medicine pack | - | - | - | - | - | - | - | - | X | X | - | - | - | X |
| Know whether repeats available on own medications | - | - | X | - | - | - | - | - | - | - | - | - | - | - |
| Able to access doctor & pharmacy | - | - | X | X† | - | - | - | - | - | - | - | - | - | - |
| Has system for taking medications when away from home | - | - | - | X† | - | X | - | - | - | - | - | - | - | - |
| Timed component or time limit | - | - | - | X | - | - | X | - | - | - | X | X | X | - |

DRUGS = Drug Regimen Unassisted Grading Scale; HMS = Hopkins Medication Schedule, MAI = Medication Assessment Instrument; MAT = Medication Administration Test; MedMaIDE = Medication Management Instrument for Deficiencies in the Elderly; MMAA = Medication Management Ability Assessment; MMEI = Medication Management Evaluation Instrument; MMPT = Medication management performance tests; MMT = Albert’s Medication Management Test; MMT-R = Albert’s Medication Management Test-Revised; MM Test = Gurland’s Medication Management Test, PA = Pharmacy Assessment; SM Task = Self-Medication Task.

X = included - = not included

* Only if relevant to patients’ current medication regimen

† Performed as part of the test, but not scored

‡ by handing pills to interviewer, placing pills into labelled boxes representing dose-times, or placing pills into a multi-compartment pillbox

§ Subject is asked to calculate number of days a supply of tablets would last, number of doses remaining when tablets removed, &/or number of doses missed based on label information and number of tablets in bottle.
