# Supplementary material for: Standardised assessment of patients' capacity to manage medications: a systematic review of published instruments
Source: BMC Geriatr. 2009 Jul 13;9:27. doi: 10.1186/1471-2318-9-27 (PMC2719637; doi:10.1186/1471-2318-9-27)
Supplement: Additional file 4 — Supplemental table S4. Reliability and validity of medication management assessment instruments. [file 1471-2318-9-27-S4.doc]

## Supplemental table 4. Reliability and validity of medication management assessment instruments

|  | **DRUGS**  [39, 53, 54, 63, 66] | **Med-Take**  [40, 62] | **Med-MaIDE** [41] | **MAI** [42, 72] | **MMEI** [43, 59, 60] | **PA** [44, 65] | **SM Task** [45] | **MM Test**  [9, 46] | **MMT**# [47, 61] | **MMT-R**#  [48] | **MMAA** [49, 53, 56-58, 64, 67] | **HMS** [50] | **MAT** [51] | **MMPT** [52] |
| --- | --- | --- | --- | --- | --- | --- | --- | --- | --- | --- | --- | --- | --- | --- |
| Validation studies |  |  |  |  |  |  |  |  |  |  |  |  |  |  |
| Sample size (primary validation sample) | 59 | 57 | 50 | 155 | 93 | 51 | 20 | 137 | 108 | 193 | 137 | 360 | 62 | 492 |
| Quality of primary validation study (out of 30) | 18 | 13 | 19 | 14 | 16 | 15 | 12 | 21 | 11 | 17 | 16 | 18 | 19 | 17 |
| No. of independent groups of investigators who have published validity data | 3 | 1 | 1 | 1 | 3 | 2 | 1 | 1 | 1 | 1 | 5 | 1 | 1 | 1 |
| Total number of patients (all studies) | 321 | 57 | 50 | 216 | 198 | 164 | 20 | 137 | 226 | 193 | >1000 | 360 | 62 | 492 |
| Reliability |  |  |  |  |  |  |  |  |  |  |  |  |  |  |
| Inter-rater reliability* | +++** |  | ++ |  |  |  |  |  | + |  |  |  |  |  |
| Test-retest reliability* | +++** |  | +++ |  |  |  |  |  |  |  | +++ |  |  |  |
| Internal consistency† |  |  | ++ |  |  |  |  | +++ | ++ | ++ |  | + |  |  |
|  |  |  |  |  |  |  |  |  |  |  |  |  |  |  |
| Content validity‡ | + | + | ++ | ++ | + | +++ | + | ++ | + | + | ++ | + | + | + |
|  |  |  |  |  |  |  |  |  |  |  |  |  |  |  |
| Construct validity§ |  |  |  |  |  |  |  |  |  |  |  |  |  |  |
| Cognitive function | ++ | ++ | ++ |  | + |  | - | +++ | + | + | ++ | + | ++ |  |
| Health literacy | + | + |  |  |  |  |  |  |  |  |  |  |  |  |
| Objectively measured medication adherence |  |  | ++ |  | - | - | - |  |  |  | +/- |  |  |  |
| Self-reported medication adherence | - |  |  | +§§ |  |  |  |  | - |  | +/- |  |  |  |
| Observed IADL performance (excluding medication management) |  |  |  |  |  |  |  |  |  |  | ++ |  | ++ |  |
| Self-reported IADL performance | - |  | - |  |  |  |  |  |  | + |  | + | ++ |  |
| Self-reported ADL performance | - |  | - |  | + |  |  |  |  |  |  |  |  |  |

|  | **DRUGS**  [39, 53, 54, 63, 66] | **Med-Take**  [40, 62] | **Med-MaIDE** [41] | **MAI** [42, 72] | **MMEI** [43, 59, 60] | **PA** [44, 65] | **SM Task** [45] | **MM Test**  [9, 46] | **MMT**# [47, 61] | **MMT-R**#  [48] | **MMAA** [49, 53, 56-58, 64, 67] | **HMS** [50] | **MAT** [51] | **MMPT** [52] |
| --- | --- | --- | --- | --- | --- | --- | --- | --- | --- | --- | --- | --- | --- | --- |
| Independent medication management | + |  |  |  | + | + |  | ++ |  |  |  |  |  | + |
| Other measure of medication management | ++ |  |  |  |  | + |  |  |  |  | ++ |  |  |  |
| Supported care residence | + |  |  |  |  |  |  |  |  |  |  |  | + |  |
| Responsiveness to change¶ | + |  |  |  |  |  |  |  |  |  | +¶¶ |  |  |  |

DRUGS = Drug Regimen Unassisted Grading Scale; HMS = Hopkins Medication Schedule, MAI = Medication Assessment Instrument; MAT = Medication Administration Test; MedMaIDE = Medication Management Instrument for Deficiencies in the Elderly; MMAA = Medication Management Ability Assessment; MMEI = Medication Management Evaluation Instrument; MMPT = Medication management performance tests; MMT = Albert’s Medication Management Test; MMT-R = Albert’s Medication Management Test-Revised; MM Test = Gurland’s Medication Management Test, PA = Pharmacy Assessment; SM Task = Self-Medication Task.

* Reliability coefficient: - < 0.5, + 0.5-0.69 (or descriptive information but no coefficient reported), ++ 0.7-0.89, +++ ≥ 0.9 [13, 19]

† Alpha coefficient: - < 0.6, + 0.6-0.69, ++ 0.7-0.79, +++ ≥ 0.8 [19]

‡ Number of medication management skill-areas assessed (see Methods section for explanation): - ≤ 2; + 3; ++ 4; +++ 5

§ Correlation coefficient: - No significant correlation; + < 0.4 (or significant but no coefficient reported); ++ 0.4-0.6; +++ > 0.6 [13, 19]

¶ Responsiveness to change: + Yes, - No

# MMT and MMT-R tested only in younger adults with Human Immunodeficiency Virus infection (mean age 39-42 years) [47, 48, 61]

** Method and number of subjects for reliability study not reported [39]

§§ Association with 2 MAI items (inability to open a flip-top lid or read a medication label); association with overall test performance not reported [42]

¶¶ Improvement in MMAA demonstrated over 12 weeks in uncontrolled pilot study (n = 16) [67]
